# Correction to Conceptual Process Design and Technoeconomic Analysis of an e-Methanol Plant with Direct Air-Captured CO_2_ and Electrolytic H_2_

**DOI:** 10.1021/acs.energyfuels.4c03268

**Published:** 2024-07-15

**Authors:** Fabio Cameli, Evangelos Delikonstantis, Afroditi Kourou, Victor Rosa, Kevin M. Van Geem, Georgios D. Stefanidis

The authors state that the open access publishing of this article is financially supported by HEAL-Link.

